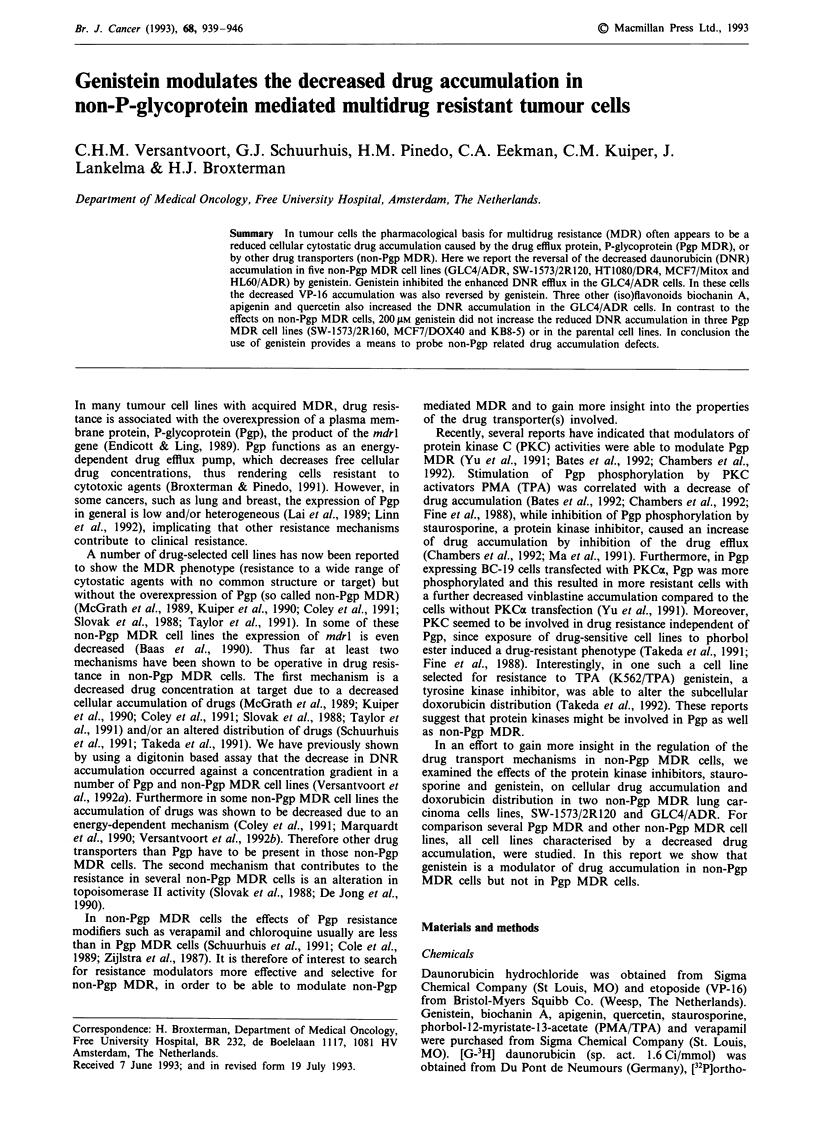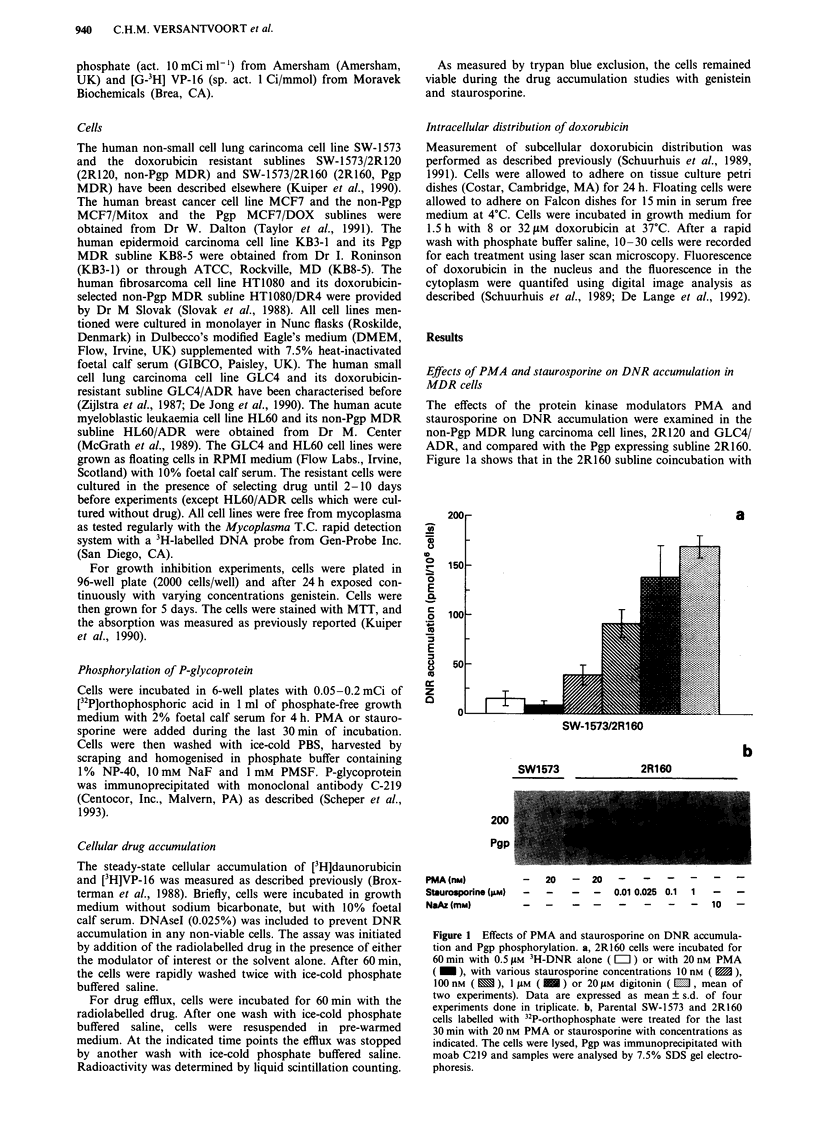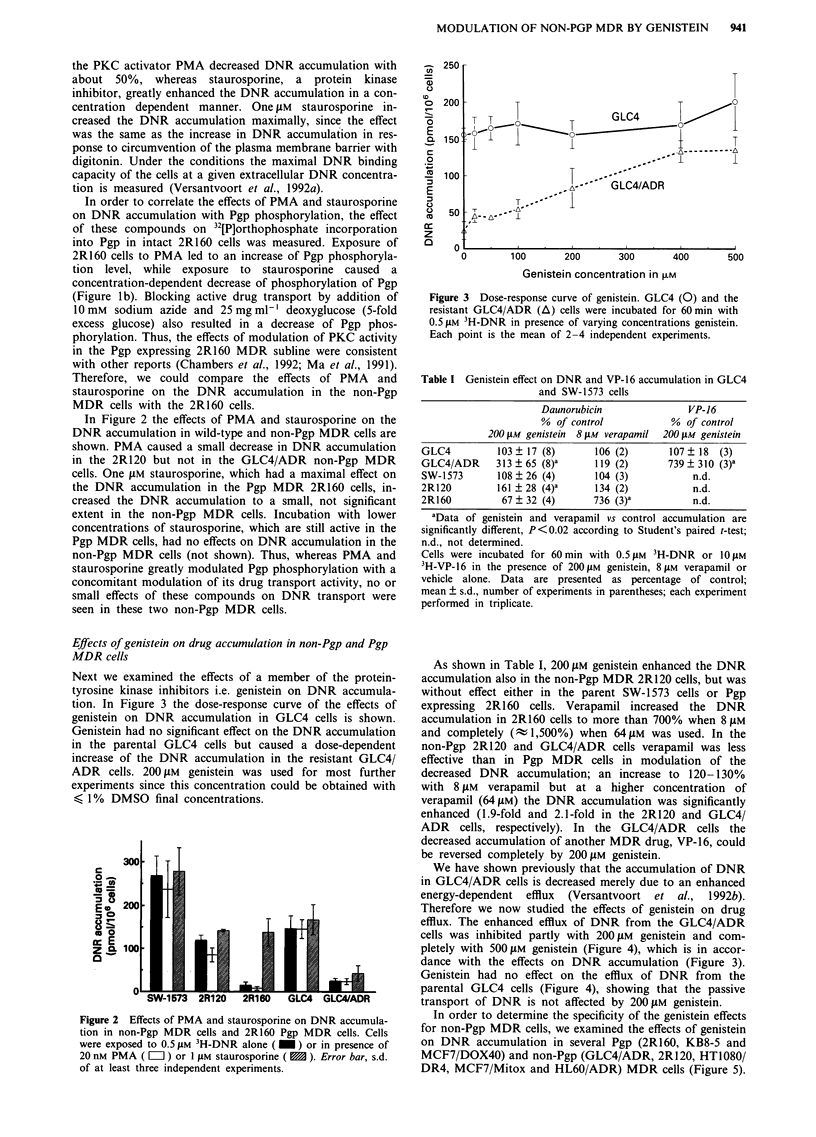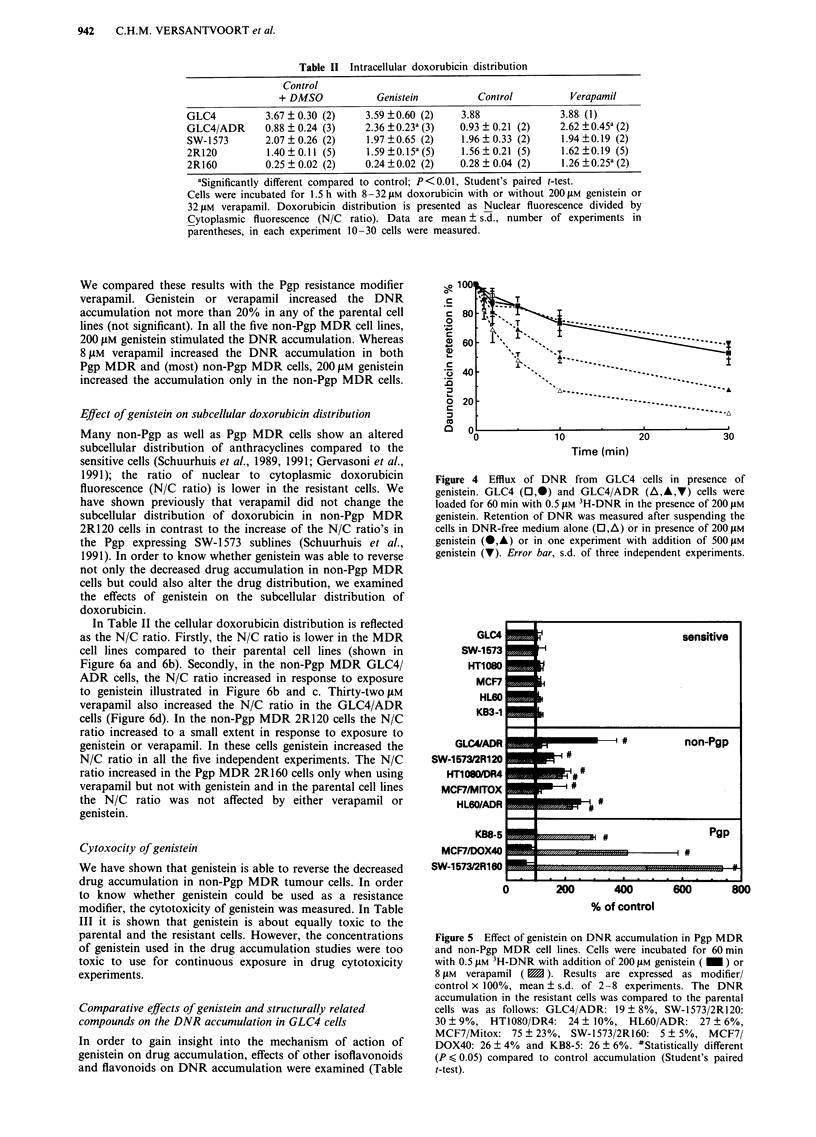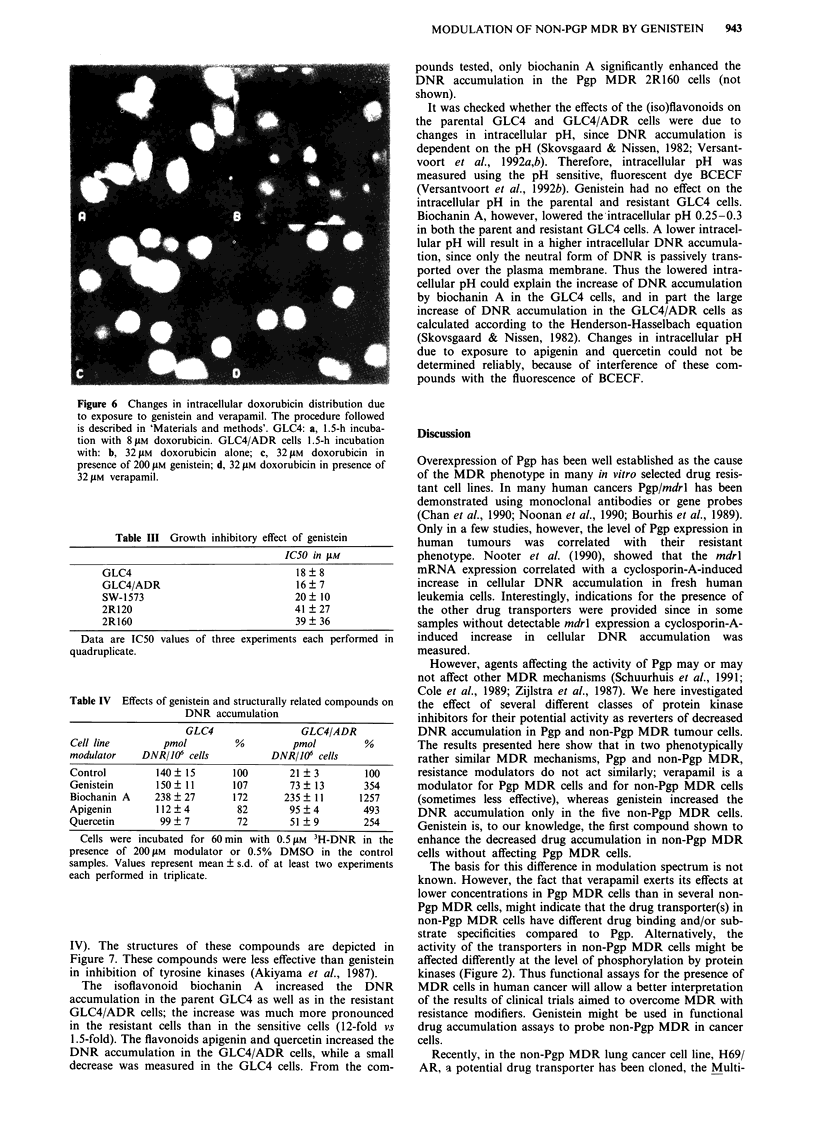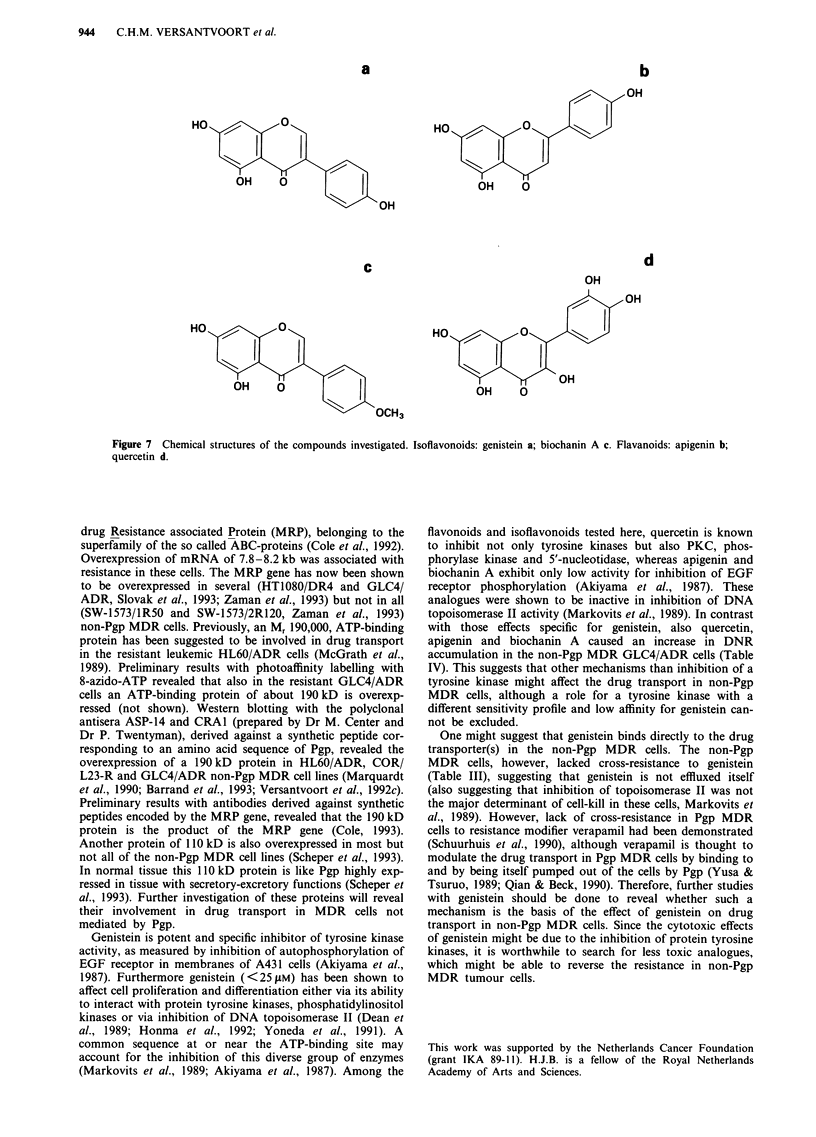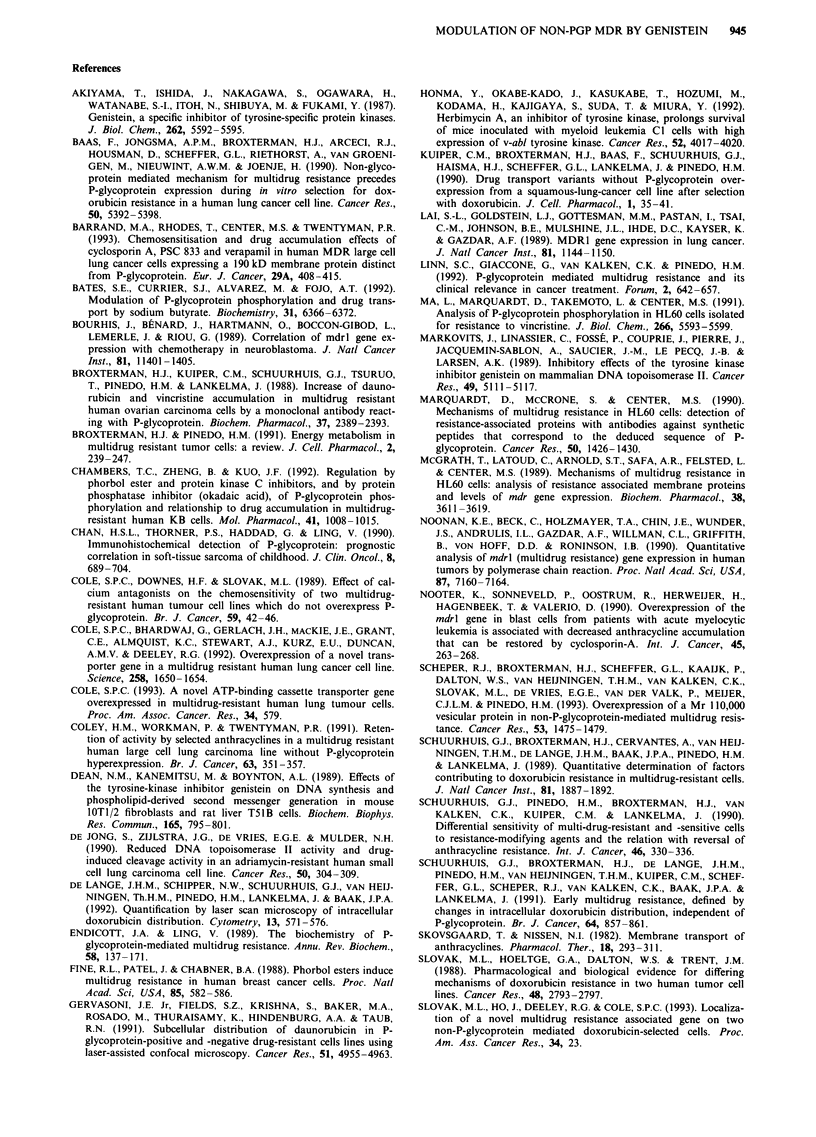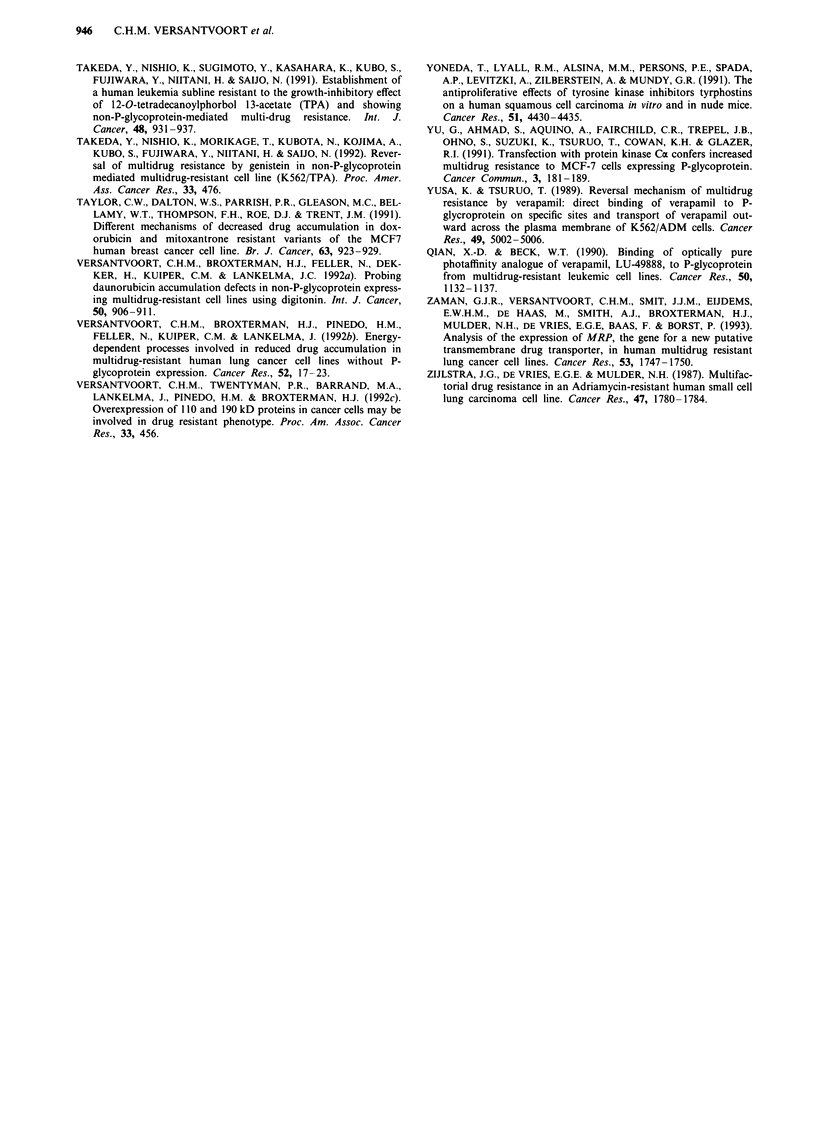# Genistein modulates the decreased drug accumulation in non-P-glycoprotein mediated multidrug resistant tumour cells.

**DOI:** 10.1038/bjc.1993.458

**Published:** 1993-11

**Authors:** C. H. Versantvoort, G. J. Schuurhuis, H. M. Pinedo, C. A. Eekman, C. M. Kuiper, J. Lankelma, H. J. Broxterman

**Affiliations:** Department of Medical Oncology, Free University Hospital, Amsterdam, The Netherlands.

## Abstract

**Images:**


### Genistein modulates the decreased drug accumulation in non-P-glycoprotein mediated multidrug resistant tumour cells

C.H.M. Versantvoort, G.J. Schuurhuis, H.M. Pinedo, C.A. Eekman, C.M. Kuiper, J. Lankelma & H.J. Broxterman

*Department of Medical Oncology, Free University Hospital, Amsterdam, The Netherlands.*

**Summary** In tumour cells the pharmacological basis for multidrug resistance (MDR) often appears to be a reduced cellular cytostatic drug accumulation caused by the drug efflux protein, P-glycoprotein (Pgp MDR), or by other drug transporters (non-Pgp MDR). Here we report the reversal of the decreased daunorubicin (DNR) accumulation in five non-Pgp MDR cell lines (GLC4/ADR, SW-1573/2R120, HT1080/DR4, MCF7/Mitox and HL60/ADR) by genistein. Genistein inhibited the enhanced DNR efflux in the GLC4/ADR cells. In these cells the decreased VP-16 accumulation was also reversed by genistein. Three other (iso)flavonoids biochanin A, apigenin and quercetin also increased the DNR accumulation in the GLC4/ADR cells. In contrast to the effects on non-Pgp MDR cells, 200 μM genistein did not increase the reduced DNR accumulation in three Pgp MDR cell lines (SW-1573/2R160, MCF7/DOX40 and KB8-5) or in the parental cell lines. In conclusion the use of genistein provides a means to probe non-Pgp related drug accumulation defects.

In many tumour cell lines with acquired MDR, drug resistance is associated with the overexpression of a plasma membrane protein, P-glycoprotein (Pgp), the product of the *mdr*l gene (Endicott & Ling, 1989). Pgp functions as an energy-dependent drug efflux pump, which decreases free cellular drug concentrations, thus rendering cells resistant to cytotoxic agents (Broxterman & Pinedo, 1991). However, in some cancers, such as lung and breast, the expression of Pgp in general is low and/or heterogeneous (Lai *et al.*, 1989; Linn *et al.*, 1992), implicating that other resistance mechanisms contribute to clinical resistance.

A number of drug-selected cell lines has now been reported to show the MDR phenotype (resistance to a wide range of cytostatic agents with no common structure or target) but without the overexpression of Pgp (so called non-Pgp MDR) (McGrath *et al.*, 1989, Kuiper *et al.*, 1990; Coley *et al.*, 1991; Slovak *et al.*, 1988; Taylor *et al.*, 1991). In some of these non-Pgp MDR cell lines the expression of *mdr*l is even decreased (Baas *et al.*, 1990). Thus far at least two mechanisms have been shown to be operative in drug resistance in non-Pgp MDR cells. The first mechanism is a decreased drug concentration at target due to a decreased cellular accumulation of drugs (McGrath *et al.*, 1989; Kuiper *et al.*, 1990; Coley *et al.*, 1991; Slovak *et al.*, 1988; Taylor *et al.*, 1991) and/or an altered distribution of drugs (Schuurhuis *et al.*, 1991; Takeda *et al.*, 1991). We have previously shown by using a digitonin based assay that the decrease in DNR accumulation occurred against a concentration gradient in a number of Pgp and non-Pgp MDR cell lines (Versantvoort *et al.*, 1992*a*). Furthermore in some non-Pgp MDR cell lines the accumulation of drugs was shown to be decreased due to an energy-dependent mechanism (Coley *et al.*, 1991; Marquardt *et al.*, 1990; Versantvoort *et al.*, 1992*b*). Therefore other drug transporters than Pgp have to be present in those non-Pgp MDR cells. The second mechanism that contributes to the resistance in several non-Pgp MDR cells is an alteration in topoisomerase II activity (Slovak *et al.*, 1988; De Jong *et al.*, 1990).

In non-Pgp MDR cells the effects of Pgp resistance modifiers such as verapamil and chloroquine usually are less than in Pgp MDR cells (Schuurhuis *et al.*, 1991; Cole *et al.*, 1989; Zijlstra *et al.*, 1987). It is therefore of interest to search for resistance modulators more effective and selective for non-Pgp MDR, in order to be able to modulate non-Pgp mediated MDR and to gain more insight into the properties of the drug transporter(s) involved.

Recently, several reports have indicated that modulators of protein kinase C (PKC) activities were able to modulate Pgp MDR (Yu *et al.*, 1991; Bates *et al.*, 1992; Chambers *et al.*, 1992). Stimulation of Pgp phosphorylation by PKC activators PMA (TPA) was correlated with a decrease of drug accumulation (Bates *et al.*, 1992; Chambers *et al.*, 1992; Fine *et al.*, 1988), while inhibition of Pgp phosphorylation by staurosporine, a protein kinase inhibitor, caused an increase of drug accumulation by inhibition of the drug efflux (Chambers *et al.*, 1992; Ma *et al.*, 1991). Furthermore, in Pgp expressing BC-19 cells transfected with PKCα, Pgp was more phosphorylated and this resulted in more resistant cells with a further decreased vinblastine accumulation compared to the cells without PKCα transfection (Yu *et al.*, 1991). Moreover, PKC seemed to be involved in drug resistance independent of Pgp, since exposure of drug-sensitive cell lines to phorbol ester induced a drug-resistant phenotype (Takeda *et al.*, 1991; Fine *et al.*, 1988). Interestingly, in one such a cell line selected for resistance to TPA (K562/TPA) genistein, a tyrosine kinase inhibitor, was able to alter the subcellular doxorubicin distribution (Takeda *et al.*, 1992). These reports suggest that protein kinases might be involved in Pgp as well as non-Pgp MDR.

In an effort to gain more insight in the regulation of the drug transport mechanisms in non-Pgp MDR cells, we examined the effects of the protein kinase inhibitors, staurosporine and genistein, on cellular drug accumulation and doxorubicin distribution in two non-Pgp MDR lung carcinoma cells lines, SW-1573/2R120 and GLC4/ADR. For comparison several Pgp MDR and other non-Pgp MDR cell lines, all cell lines characterised by a decreased drug accumulation, were studied. In this report we show that genistein is a modulator of drug accumulation in non-Pgp MDR cells but not in Pgp MDR cells.

### Materials and methods

### Chemicals

Daunorubicin hydrochloride was obtained from Sigma Chemical Company (St Louis, MO) and etoposide (VP-16) from Bristol-Myers Squibb Co. (Weesp, The Netherlands). Genistein, biochanin A, apigenin, quercetin, staurosporine, phorbol-12-myristate-13-acetate (PMA/TPA) and verapamil were purchased from Sigma Chemical Company (St. Louis, MO). [G-$^3$H] daunorubicin (sp. act. 1.6 Ci/mmol) was obtained from Du Pont de Neumours (Germany), [$^{32}$P]ortho-

Correspondence: H. Broxterman, Department of Medical Oncology, Free University Hospital, BR 232, de Boelelaan 1117, 1081 HV Amsterdam, The Netherlands.
Received 7 June 1993; and in revised form 19 July 1993.

phosphate (act. 10 mCi ml$^{-1}$) from Amersham (Amersham, UK) and [G-$^3$H] VP-16 (sp. act. 1 Ci/mmol) from Moravek Biochemicals (Brea, CA).

*Cells*

The human non-small cell lung carincoma cell line SW-1573 and the doxorubicin resistant sublines SW-1573/2R120 (2R120, non-Pgp MDR) and SW-1573/2R160 (2R160, Pgp MDR) have been described elsewhere (Kuiper *et al.*, 1990). The human breast cancer cell line MCF7 and the non-Pgp MCF7/Mitox and the Pgp MCF7/DOX sublines were obtained from Dr W. Dalton (Taylor *et al.*, 1991). The human epidermoid carcinoma cell line KB3-1 and its Pgp MDR subline KB8-5 were obtained from Dr I. Roninson (KB3-1) or through ATCC, Rockville, MD (KB8-5). The human fibrosarcoma cell line HT1080 and its doxorubicin-selected non-Pgp MDR subline HT1080/DR4 were provided by Dr M Slovak (Slovak *et al.*, 1988). All cell lines mentioned were cultured in monolayer in Nunc flasks (Roskilde, Denmark) in Dulbecco's modified Eagle's medium (DMEM, Flow, Irvine, UK) supplemented with 7.5% heat-inactivated foetal calf serum (GIBCO, Paisley, UK). The human small cell lung carcinoma cell line GLC4 and its doxorubicin-resistant subline GLC4/ADR have been characterised before (Zijlstra *et al.*, 1987; De Jong *et al.*, 1990). The human acute myeloblastic leukaemia cell line HL60 and its non-Pgp MDR subline HL60/ADR were obtained from Dr M. Center (McGrath *et al.*, 1989). The GLC4 and HL60 cell lines were grown as floating cells in RPMI medium (Flow Labs., Irvine, Scotland) with 10% foetal calf serum. The resistant cells were cultured in the presence of selecting drug until 2–10 days before experiments (except HL60/ADR cells which were cultured without drug). All cell lines were free from mycoplasma as tested regularly with the *Mycoplasma* T.C. rapid detection system with a $^3$H-labelled DNA probe from Gen-Probe Inc. (San Diego, CA).

For growth inhibition experiments, cells were plated in 96-well plate (2000 cells/well) and after 24 h exposed continuously with varying concentrations genistein. Cells were then grown for 5 days. The cells were stained with MTT, and the absorption was measured as previously reported (Kuiper *et al.*, 1990).

*Phosphorylation of P-glycoprotein*

Cells were incubated in 6-well plates with 0.05–0.2 mCi of [$^{32}$P]orthophosphoric acid in 1 ml of phosphate-free growth medium with 2% foetal calf serum for 4 h. PMA or staurosporine were added during the last 30 min of incubation. Cells were then washed with ice-cold PBS, harvested by scraping and homogenised in phosphate buffer containing 1% NP-40, 10 mM NaF and 1 mM PMSF. P-glycoprotein was immunoprecipitated with monoclonal antibody C-219 (Centocor, Inc., Malvern, PA) as described (Scheper *et al.*, 1993).

*Cellular drug accumulation*

The steady-state cellular accumulation of [$^3$H]daunorubicin and [$^3$H]VP-16 was measured as described previously (Broxterman *et al.*, 1988). Briefly, cells were incubated in growth medium without sodium bicarbonate, but with 10% foetal calf serum. DNAseI (0.025%) was included to prevent DNR accumulation in any non-viable cells. The assay was initiated by addition of the radiolabelled drug in the presence of either the modulator of interest or the solvent alone. After 60 min, the cells were rapidly washed twice with ice-cold phosphate buffered saline.

For drug efflux, cells were incubated for 60 min with the radiolabelled drug. After one wash with ice-cold phosphate buffered saline, cells were resuspended in pre-warmed medium. At the indicated time points the efflux was stopped by another wash with ice-cold phosphate buffered saline. Radioactivity was determined by liquid scintillation counting.

As measured by trypan blue exclusion, the cells remained viable during the drug accumulation studies with genistein and staurosporine.

*Intracellular distribution of doxorubicin*

Measurement of subcellular doxorubicin distribution was performed as described previously (Schuurhuis *et al.*, 1989, 1991). Cells were allowed to adhere on tissue culture petri dishes (Costar, Cambridge, MA) for 24 h. Floating cells were allowed to adhere on Falcon dishes for 15 min in serum free medium at 4°C. Cells were incubated in growth medium for 1.5 h with 8 or 32 $\mu$M doxorubicin at 37°C. After a rapid wash with phosphate buffer saline, 10–30 cells were recorded for each treatment using laser scan microscopy. Fluorescence of doxorubicin in the nucleus and the fluorescence in the cytoplasm were quantifed using digital image analysis as described (Schuurhuis *et al.*, 1989; De Lange *et al.*, 1992).

### Results

*Effects of PMA and staurosporine on DNR accumulation in MDR cells*

The effects of the protein kinase modulators PMA and staurosporine on DNR accumulation were examined in the non-Pgp MDR lung carcinoma cell lines, 2R120 and GLC4/ADR, and compared with the Pgp expressing subline 2R160. Figure 1a shows that in the 2R160 subline coincubation with

**Figure 1** Effects of PMA and staurosporine on DNR accumulation and Pgp phosphorylation. **a**, 2R160 cells were incubated for 60 min with 0.5 $\mu$M $^3$H-DNR alone (□) or with 20 nM PMA (■), with various staurosporine concentrations 10 nM (▨), 100 nM (▧), 1 $\mu$M (▩) or 20 $\mu$M digitonin (▒, mean of two experiments). Data are expressed as mean ± s.d. of four experiments done in triplicate. **b**, Parental SW-1573 and 2R160 cells labelled with $^{32}$P-orthophosphate were treated for the last 30 min with 20 nM PMA or staurosporine with concentrations as indicated. The cells were lysed, Pgp was immunoprecipitated with moab C219 and samples were analysed by 7.5% SDS gel electrophoresis.

the PKC activator PMA decreased DNR accumulation with about 50%, whereas staurosporine, a protein kinase inhibitor, greatly enhanced the DNR accumulation in a concentration dependent manner. One μM staurosporine increased the DNR accumulation maximally, since the effect was the same as the increase in DNR accumulation in response to circumvention of the plasma membrane barrier with digitonin. Under the conditions the maximal DNR binding capacity of the cells at a given extracellular DNR concentration is measured (Versantvoort *et al.*, 1992*a*).

In order to correlate the effects of PMA and staurosporine on DNR accumulation with Pgp phosphorylation, the effect of these compounds on $^{32}$[P]orthophosphate incorporation into Pgp in intact 2R160 cells was measured. Exposure of 2R160 cells to PMA led to an increase of Pgp phosphorylation level, while exposure to staurosporine caused a concentration-dependent decrease of phosphorylation of Pgp (Figure 1b). Blocking active drug transport by addition of 10 mM sodium azide and 25 mg ml$^{-1}$ deoxyglucose (5-fold excess glucose) also resulted in a decrease of Pgp phosphorylation. Thus, the effects of modulation of PKC activity in the Pgp expressing 2R160 MDR subline were consistent with other reports (Chambers *et al.*, 1992; Ma *et al.*, 1991). Therefore, we could compare the effects of PMA and staurosporine on the DNR accumulation in the non-Pgp MDR cells with the 2R160 cells.

In Figure 2 the effects of PMA and staurosporine on the DNR accumulation in wild-type and non-Pgp MDR cells are shown. PMA caused a small decrease in DNR accumulation in the 2R120 but not in the GLC4/ADR non-Pgp MDR cells. One μM staurosporine, which had a maximal effect on the DNR accumulation in the Pgp MDR 2R160 cells, increased the DNR accumulation to a small, not significant extent in the non-Pgp MDR cells. Incubation with lower concentrations of staurosporine, which are still active in the Pgp MDR cells, had no effects on DNR accumulation in the non-Pgp MDR cells (not shown). Thus, whereas PMA and staurosporine greatly modulated Pgp phosphorylation with a concomitant modulation of its drug transport activity, no or small effects of these compounds on DNR transport were seen in these two non-Pgp MDR cells.

*Effects of genistein on drug accumulation in non-Pgp and Pgp MDR cells*

Next we examined the effects of a member of the protein-tyrosine kinase inhibitors i.e. genistein on DNR accumulation. In Figure 3 the dose-response curve of the effects of genistein on DNR accumulation in GLC4 cells is shown. Genistein had no significant effect on the DNR accumulation in the parental GLC4 cells but caused a dose-dependent increase of the DNR accumulation in the resistant GLC4/ADR cells. 200 μM genistein was used for most further experiments since this concentration could be obtained with ⩽ 1% DMSO final concentrations.

**Figure 2** Effects of PMA and staurosporine on DNR accumulation in non-Pgp MDR cells and 2R160 Pgp MDR cells. Cells were exposed to 0.5 μM $^3$H-DNR alone (■) or in presence of 20 nM PMA (□) or 1 μM staurosporine (▨). *Error bar*, s.d. of at least three independent experiments.

**Figure 3** Dose-response curve of genistein. GLC4 (○) and the resistant GLC4/ADR (△) cells were incubated for 60 min with 0.5 μM $^3$H-DNR in presence of varying concentrations genistein. Each point is the mean of 2–4 independent experiments.

**Table I** Genistein effect on DNR and VP-16 accumulation in GLC4 and SW-1573 cells

| | *Daunorubicin % of control* | | *VP-16 % of control* |
|---|---|---|---|
| | *200 μM genistein* | *8 μM verapamil* | *200 μM genistein* |
| GLC4 | 103 ± 17 (8) | 106 (2) | 107 ± 18 (3) |
| GLC4/ADR | 313 ± 65 (8)[a] | 119 (2) | 739 ± 310 (3)[a] |
| SW-1573 | 108 ± 26 (4) | 104 (3) | n.d. |
| 2R120 | 161 ± 28 (4)[a] | 134 (2) | n.d. |
| 2R160 | 67 ± 32 (4) | 736 (3)[a] | n.d. |

[a]Data of genistein and verapamil *vs* control accumulation are significantly different, $P < 0.02$ according to Student's paired *t*-test; n.d., not determined.
Cells were incubated for 60 min with 0.5 μM $^3$H-DNR or 10 μM $^3$H-VP-16 in the presence of 200 μM genistein, 8 μM verapamil or vehicle alone. Data are presented as percentage of control; mean ± s.d., number of experiments in parentheses; each experiment performed in triplicate.

As shown in Table I, 200 μM genistein enhanced the DNR accumulation also in the non-Pgp MDR 2R120 cells, but was without effect either in the parent SW-1573 cells or Pgp expressing 2R160 cells. Verapamil increased the DNR accumulation in 2R160 cells to more than 700% when 8 μM and completely (≈1,500%) when 64 μM was used. In the non-Pgp 2R120 and GLC4/ADR cells verapamil was less effective than in Pgp MDR cells in modulation of the decreased DNR accumulation; an increase to 120–130% with 8 μM verapamil but at a higher concentration of verapamil (64 μM) the DNR accumulation was significantly enhanced (1.9-fold and 2.1-fold in the 2R120 and GLC4/ADR cells, respectively). In the GLC4/ADR cells the decreased accumulation of another MDR drug, VP-16, could be reversed completely by 200 μM genistein.

We have shown previously that the accumulation of DNR in GLC4/ADR cells is decreased merely due to an enhanced energy-dependent efflux (Versantvoort *et al.*, 1992*b*). Therefore we now studied the effects of genistein on drug efflux. The enhanced efflux of DNR from the GLC4/ADR cells was inhibited partly with 200 μM genistein and completely with 500 μM genistein (Figure 4), which is in accordance with the effects on DNR accumulation (Figure 3). Genistein had no effect on the efflux of DNR from the parental GLC4 cells (Figure 4), showing that the passive transport of DNR is not affected by 200 μM genistein.

In order to determine the specificity of the genistein effects for non-Pgp MDR cells, we examined the effects of genistein on DNR accumulation in several Pgp (2R160, KB8-5 and MCF7/DOX40) and non-Pgp (GLC4/ADR, 2R120, HT1080/DR4, MCF7/Mitox and HL60/ADR) MDR cells (Figure 5).

**Table II** Intracellular doxorubicin distribution

| | *Control + DMSO* | *Genistein* | *Control* | *Verapamil* |
|---|---|---|---|---|
| GLC4 | 3.67 ± 0.30 (2) | 3.59 ± 0.60 (2) | 3.88 | 3.88 (1) |
| GLC4/ADR | 0.88 ± 0.24 (3) | 2.36 ± 0.23[a] (3) | 0.93 ± 0.21 (2) | 2.62 ± 0.45[a] (2) |
| SW-1573 | 2.07 ± 0.26 (2) | 1.97 ± 0.65 (2) | 1.96 ± 0.33 (2) | 1.94 ± 0.19 (2) |
| 2R120 | 1.40 ± 0.11 (5) | 1.59 ± 0.15[a] (5) | 1.56 ± 0.21 (5) | 1.62 ± 0.19 (5) |
| 2R160 | 0.25 ± 0.02 (2) | 0.24 ± 0.02 (2) | 0.28 ± 0.04 (2) | 1.26 ± 0.25[a] (2) |

[a]Significantly different compared to control; $P < 0.01$, Student's paired *t*-test.
Cells were incubated for 1.5 h with 8–32 μM doxorubicin with or without 200 μM genistein or 32 μM verapamil. Doxorubicin distribution is presented as Nuclear fluorescence divided by Cytoplasmic fluorescence (N/C ratio). Data are mean ± s.d., number of experiments in parentheses, in each experiment 10–30 cells were measured.

We compared these results with the Pgp resistance modifier verapamil. Genistein or verapamil increased the DNR accumulation not more than 20% in any of the parental cell lines (not significant). In all the five non-Pgp MDR cell lines, 200 μM genistein stimulated the DNR accumulation. Whereas 8 μM verapamil increased the DNR accumulation in both Pgp MDR and (most) non-Pgp MDR cells, 200 μM genistein increased the accumulation only in the non-Pgp MDR cells.

*Effect of genistein on subcellular doxorubicin distribution*

Many non-Pgp as well as Pgp MDR cells show an altered subcellular distribution of anthracyclines compared to the sensitive cells (Schuurhuis *et al.*, 1989, 1991; Gervasoni *et al.*, 1991); the ratio of nuclear to cytoplasmic doxorubicin fluorescence (N/C ratio) is lower in the resistant cells. We have shown previously that verapamil did not change the subcellular distribution of doxorubicin in non-Pgp MDR 2R120 cells in contrast to the increase of the N/C ratio's in the Pgp expressing SW-1573 sublines (Schuurhuis *et al.*, 1991). In order to know whether genistein was able to reverse not only the decreased drug accumulation in non-Pgp MDR cells but could also alter the drug distribution, we examined the effects of genistein on the subcellular distribution of doxorubicin.

In Table II the cellular doxorubicin distribution is reflected as the N/C ratio. Firstly, the N/C ratio is lower in the MDR cell lines compared to their parental cell lines (shown in Figure 6a and 6b). Secondly, in the non-Pgp MDR GLC4/ADR cells, the N/C ratio increased in response to exposure to genistein illustrated in Figure 6b and c. Thirty-two μM verapamil also increased the N/C ratio in the GLC4/ADR cells (Figure 6d). In the non-Pgp MDR 2R120 cells the N/C ratio increased to a small extent in response to exposure to genistein or verapamil. In these cells genistein increased the N/C ratio in all the five independent experiments. The N/C ratio increased in the Pgp MDR 2R160 cells only when using verapamil but not with genistein and in the parental cell lines the N/C ratio was not affected by either verapamil or genistein.

*Cytoxocity of genistein*

We have shown that genistein is able to reverse the decreased drug accumulation in non-Pgp MDR tumour cells. In order to know whether genistein could be used as a resistance modifier, the cytotoxicity of genistein was measured. In Table III it is shown that genistein is about equally toxic to the parental and the resistant cells. However, the concentrations of genistein used in the drug accumulation studies were too toxic to use for continuous exposure in drug cytotoxicity experiments.

*Comparative effects of genistein and structurally related compounds on the DNR accumulation in GLC4 cells*

In order to gain insight into the mechanism of action of genistein on drug accumulation, effects of other isoflavonoids and flavonoids on DNR accumulation were examined (Table

**Figure 4** Efflux of DNR from GLC4 cells in presence of genistein. GLC4 (□,●) and GLC4/ADR (△,▲,▼) cells were loaded for 60 min with 0.5 μM $^3$H-DNR in the presence of 200 μM genistein. Retention of DNR was measured after suspending the cells in DNR-free medium alone (□,△) or in presence of 200 μM genistein (●,▲) or in one experiment with addition of 500 μM genistein (▼). *Error bar*, s.d. of three independent experiments.

**Figure 5** Effect of genistein on DNR accumulation in Pgp MDR and non-Pgp MDR cell lines. Cells were incubated for 60 min with 0.5 μM $^3$H-DNR with addition of 200 μM genistein (■) or 8 μM verapamil (▨). Results are expressed as modifier/control × 100%, mean ± s.d. of 2–8 experiments. The DNR accumulation in the resistant cells was compared to the parental cells was as follows: GLC4/ADR: 19 ± 8%, SW-1573/2R120: 30 ± 9%, HT1080/DR4: 24 ± 10%, HL60/ADR: 27 ± 6%, MCF7/Mitox: 75 ± 23%, SW-1573/2R160: 5 ± 5%, MCF7/DOX40: 26 ± 4% and KB8-5: 26 ± 6%. #Statistically different ($P \leq 0.05$) compared to control accumulation (Student's paired *t*-test).

**Figure 6** Changes in intracellular doxorubicin distribution due to exposure to genistein and verapamil. The procedure followed is described in 'Materials and methods'. GLC4: **a**, 1.5-h incubation with 8 μM doxorubicin. GLC4/ADR cells 1.5-h incubation with: **b**, 32 μM doxorubicin alone; **c**, 32 μM doxorubicin in presence of 200 μM genistein; **d**, 32 μM doxorubicin in presence of 32 μM verapamil.

**Table III** Growth inhibitory effect of genistein

| | *IC50 in μM* |
|---|---|
| GLC4 | 18 ± 8 |
| GLC4/ADR | 16 ± 7 |
| SW-1573 | 20 ± 10 |
| 2R120 | 41 ± 27 |
| 2R160 | 39 ± 36 |

Data are IC50 values of three experiments each performed in quadruplicate.

**Table IV** Effects of genistein and structurally related compounds on DNR accumulation

| *Cell line modulator* | *GLC4 pmol DNR/10⁶ cells* | *%* | *GLC4/ADR pmol DNR/10⁶ cells* | *%* |
|---|---|---|---|---|
| Control | 140 ± 15 | 100 | 21 ± 3 | 100 |
| Genistein | 150 ± 11 | 107 | 73 ± 13 | 354 |
| Biochanin A | 238 ± 27 | 172 | 235 ± 11 | 1257 |
| Apigenin | 112 ± 4 | 82 | 95 ± 4 | 493 |
| Quercetin | 99 ± 7 | 72 | 51 ± 9 | 254 |

Cells were incubated for 60 min with 0.5 μM $^3$H-DNR in the presence of 200 μM modulator or 0.5% DMSO in the control samples. Values represent mean ± s.d. of at least two experiments each performed in triplicate.

IV). The structures of these compounds are depicted in Figure 7. These compounds were less effective than genistein in inhibition of tyrosine kinases (Akiyama *et al.*, 1987).

The isoflavonoid biochanin A increased the DNR accumulation in the parent GLC4 as well as in the resistant GLC4/ADR cells; the increase was much more pronounced in the resistant cells than in the sensitive cells (12-fold *vs* 1.5-fold). The flavonoids apigenin and quercetin increased the DNR accumulation in the GLC4/ADR cells, while a small decrease was measured in the GLC4 cells. From the compounds tested, only biochanin A significantly enhanced the DNR accumulation in the Pgp MDR 2R160 cells (not shown).

It was checked whether the effects of the (iso)flavonoids on the parental GLC4 and GLC4/ADR cells were due to changes in intracellular pH, since DNR accumulation is dependent on the pH (Skovsgaard & Nissen, 1982; Versantvoort *et al.*, 1992*a*,*b*). Therefore, intracellular pH was measured using the pH sensitive, fluorescent dye BCECF (Versantvoort *et al.*, 1992*b*). Genistein had no effect on the intracellular pH in the parental and resistant GLC4 cells. Biochanin A, however, lowered the intracellular pH 0.25–0.3 in both the parent and resistant GLC4 cells. A lower intracellular pH will result in a higher intracellular DNR accumulation, since only the neutral form of DNR is passively transported over the plasma membrane. Thus the lowered intracellular pH could explain the increase of DNR accumulation by biochanin A in the GLC4 cells, and in part the large increase of DNR accumulation in the GLC4/ADR cells as calculated according to the Henderson-Hasselbach equation (Skovsgaard & Nissen, 1982). Changes in intracellular pH due to exposure to apigenin and quercetin could not be determined reliably, because of interference of these compounds with the fluorescence of BCECF.

### Discussion

Overexpression of Pgp has been well established as the cause of the MDR phenotype in many *in vitro* selected drug resistant cell lines. In many human cancers Pgp/*mdr*l has been demonstrated using monoclonal antibodies or gene probes (Chan *et al.*, 1990; Noonan *et al.*, 1990; Bourhis *et al.*, 1989). Only in a few studies, however, the level of Pgp expression in human tumours was correlated with their resistant phenotype. Nooter *et al.* (1990), showed that the *mdr*l mRNA expression correlated with a cyclosporin-A-induced increase in cellular DNR accumulation in fresh human leukemia cells. Interestingly, indications for the presence of the other drug transporters were provided since in some samples without detectable *mdr*l expression a cyclosporin-A-induced increase in cellular DNR accumulation was measured.

However, agents affecting the activity of Pgp may or may not affect other MDR mechanisms (Schuurhuis *et al.*, 1991; Cole *et al.*, 1989; Zijlstra *et al.*, 1987). We here investigated the effect of several different classes of protein kinase inhibitors for their potential activity as reverters of decreased DNR accumulation in Pgp and non-Pgp MDR tumour cells. The results presented here show that in two phenotypically rather similar MDR mechanisms, Pgp and non-Pgp MDR, resistance modulators do not act similarly; verapamil is a modulator for Pgp MDR cells and for non-Pgp MDR cells (sometimes less effective), whereas genistein increased the DNR accumulation only in the five non-Pgp MDR cells. Genistein is, to our knowledge, the first compound shown to enhance the decreased drug accumulation in non-Pgp MDR cells without affecting Pgp MDR cells.

The basis for this difference in modulation spectrum is not known. However, the fact that verapamil exerts its effects at lower concentrations in Pgp MDR cells than in several non-Pgp MDR cells, might indicate that the drug transporter(s) in non-Pgp MDR cells have different drug binding and/or substrate specificities compared to Pgp. Alternatively, the activity of the transporters in non-Pgp MDR cells might be affected differently at the level of phosphorylation by protein kinases (Figure 2). Thus functional assays for the presence of MDR cells in human cancer will allow a better interpretation of the results of clinical trials aimed to overcome MDR with resistance modifiers. Genistein might be used in functional drug accumulation assays to probe non-Pgp MDR in cancer cells.

Recently, in the non-Pgp MDR lung cancer cell line, H69/AR, a potential drug transporter has been cloned, the Multi-

**Figure 7** Chemical structures of the compounds investigated. Isoflavonoids: genistein **a**; biochanin A **c**. Flavanoids: apigenin **b**; quercetin **d**.

drug Resistance associated Protein (MRP), belonging to the superfamily of the so called ABC-proteins (Cole *et al.*, 1992). Overexpression of mRNA of 7.8–8.2 kb was associated with resistance in these cells. The MRP gene has now been shown to be overexpressed in several (HT1080/DR4 and GLC4/ADR, Slovak *et al.*, 1993; Zaman *et al.*, 1993) but not in all (SW-1573/1R50 and SW-1573/2R120, Zaman *et al.*, 1993) non-Pgp MDR cells. Previously, an $M_r$ 190,000, ATP-binding protein has been suggested to be involved in drug transport in the resistant leukemic HL60/ADR cells (McGrath *et al.*, 1989). Preliminary results with photoaffinity labelling with 8-azido-ATP revealed that also in the resistant GLC4/ADR cells an ATP-binding protein of about 190 kD is overexpressed (not shown). Western blotting with the polyclonal antisera ASP-14 and CRA1 (prepared by Dr M. Center and Dr P. Twentyman), derived against a synthetic peptide corresponding to an amino acid sequence of Pgp, revealed the overexpression of a 190 kD protein in HL60/ADR, COR/L23-R and GLC4/ADR non-Pgp MDR cell lines (Marquardt *et al.*, 1990; Barrand *et al.*, 1993; Versantvoort *et al.*, 1992*c*). Preliminary results with antibodies derived against synthetic peptides encoded by the MRP gene, revealed that the 190 kD protein is the product of the MRP gene (Cole, 1993). Another protein of 110 kD is also overexpressed in most but not all of the non-Pgp MDR cell lines (Scheper *et al.*, 1993). In normal tissue this 110 kD protein is like Pgp highly expressed in tissue with secretory-excretory functions (Scheper *et al.*, 1993). Further investigation of these proteins will reveal their involvement in drug transport in MDR cells not mediated by Pgp.

Genistein is potent and specific inhibitor of tyrosine kinase activity, as measured by inhibition of autophosphorylation of EGF receptor in membranes of A431 cells (Akiyama *et al.*, 1987). Furthermore genistein ($<25\,\mu$M) has been shown to affect cell proliferation and differentiation either via its ability to interact with protein tyrosine kinases, phosphatidylinositol kinases or via inhibition of DNA topoisomerase II (Dean *et al.*, 1989; Honma *et al.*, 1992; Yoneda *et al.*, 1991). A common sequence at or near the ATP-binding site may account for the inhibition of this diverse group of enzymes (Markovits *et al.*, 1989; Akiyama *et al.*, 1987). Among the flavonoids and isoflavonoids tested here, quercetin is known to inhibit not only tyrosine kinases but also PKC, phosphorylase kinase and 5′-nucleotidase, whereas apigenin and biochanin A exhibit only low activity for inhibition of EGF receptor phosphorylation (Akiyama *et al.*, 1987). These analogues were shown to be inactive in inhibition of DNA topoisomerase II activity (Markovits *et al.*, 1989). In contrast with those effects specific for genistein, also quercetin, apigenin and biochanin A caused an increase in DNR accumulation in the non-Pgp MDR GLC4/ADR cells (Table IV). This suggests that other mechanisms than inhibition of a tyrosine kinase might affect the drug transport in non-Pgp MDR cells, although a role for a tyrosine kinase with a different sensitivity profile and low affinity for genistein cannot be excluded.

One might suggest that genistein binds directly to the drug transporter(s) in the non-Pgp MDR cells. The non-Pgp MDR cells, however, lacked cross-resistance to genistein (Table III), suggesting that genistein is not effluxed itself (also suggesting that inhibition of topoisomerase II was not the major determinant of cell-kill in these cells, Markovits *et al.*, 1989). However, lack of cross-resistance in Pgp MDR cells to resistance modifier verapamil had been demonstrated (Schuurhuis *et al.*, 1990), although verapamil is thought to modulate the drug transport in Pgp MDR cells by binding to and by being itself pumped out of the cells by Pgp (Yusa & Tsuruo, 1989; Qian & Beck, 1990). Therefore, further studies with genistein should be done to reveal whether such a mechanism is the basis of the effect of genistein on drug transport in non-Pgp MDR cells. Since the cytotoxic effects of genistein might be due to the inhibition of protein tyrosine kinases, it is worthwhile to search for less toxic analogues, which might be able to reverse the resistance in non-Pgp MDR tumour cells.

This work was supported by the Netherlands Cancer Foundation (grant IKA 89-11). H.J.B. is a fellow of the Royal Netherlands Academy of Arts and Sciences.

### References

AKIYAMA, T., ISHIDA, J., NAKAGAWA, S., OGAWARA, H., WATANABE, S.-I., ITOH, N., SHIBUYA, M. & FUKAMI, Y. (1987). Genistein, a specific inhibitor of tyrosine-specific protein kinases. *J. Biol. Chem.*, **262**, 5592–5595.

BAAS, F., JONGSMA, A.P.M., BROXTERMAN, H.J., ARCECI, R.J., HOUSMAN, D., SCHEFFER, G.L., RIETHORST, A., VAN GROENIGEN, M., NIEUWINT, A.W.M. & JOENJE, H. (1990). Non-glycoprotein mediated mechanism for multidrug resistance precedes P-glycoprotein expression during *in vitro* selection for doxorubicin resistance in a human lung cancer cell line. *Cancer Res.*, **50**, 5392–5398.

BARRAND, M.A., RHODES, T., CENTER, M.S. & TWENTYMAN, P.R. (1993). Chemosensitisation and drug accumulation effects of cyclosporin A, PSC 833 and verapamil in human MDR large cell lung cancer cells expressing a 190 kD membrane protein distinct from P-glycoprotein. *Eur. J. Cancer*, **29A**, 408–415.

BATES, S.E., CURRIER, S.J., ALVAREZ, M. & FOJO, A.T. (1992). Modulation of P-glycoprotein phosphorylation and drug transport by sodium butyrate. *Biochemistry*, **31**, 6366–6372.

BOURHIS, J., BÉNARD, J., HARTMANN, O., BOCCON-GIBOD, L., LEMERLE, J. & RIOU, G. (1989). Correlation of mdr1 gene expression with chemotherapy in neuroblastoma. *J. Natl Cancer Inst.*, **81**, 11401–1405.

BROXTERMAN, H.J., KUIPER, C.M., SCHUURHUIS, G.J., TSURUO, T., PINEDO, H.M. & LANKELMA, J. (1988). Increase of daunorubicin and vincristine accumulation in multidrug resistant human ovarian carcinoma cells by a monoclonal antibody reacting with P-glycoprotein. *Biochem. Pharmacol.*, **37**, 2389–2393.

BROXTERMAN, H.J. & PINEDO, H.M. (1991). Energy metabolism in multidrug resistant tumor cells: a review. *J. Cell. Pharmacol.*, **2**, 239–247.

CHAMBERS, T.C., ZHENG, B. & KUO, J.F. (1992). Regulation by phorbol ester and protein kinase C inhibitors, and by protein phosphatase inhibitor (okadaic acid), of P-glycoprotein phosphorylation and relationship to drug accumulation in multidrug-resistant human KB cells. *Mol. Pharmacol.*, **41**, 1008–1015.

CHAN, H.S.L., THORNER, P.S., HADDAD, G. & LING, V. (1990). Immunohistochemical detection of P-glycoprotein: prognostic correlation in soft-tissue sarcoma of childhood. *J. Clin. Oncol.*, **8**, 689–704.

COLE, S.P.C., DOWNES, H.F. & SLOVAK, M.L. (1989). Effect of calcium antagonists on the chemosensitivity of two multidrug-resistant human tumour cell lines which do not overexpress P-glycoprotein. *Br. J. Cancer*, **59**, 42–46.

COLE, S.P.C., BHARDWAJ, G., GERLACH, J.H., MACKIE, J.E., GRANT, C.E., ALMQUIST, K.C., STEWART, A.J., KURZ, E.U., DUNCAN, A.M.V. & DEELEY, R.G. (1992). Overexpression of a novel transporter gene in a multidrug resistant human lung cancer cell line. *Science*, **258**, 1650–1654.

COLE, S.P.C. (1993). A novel ATP-binding cassette transporter gene overexpressed in multidrug-resistant human lung tumour cells. *Proc. Am. Assoc. Cancer. Res.*, **34**, 579.

COLEY, H.M., WORKMAN, P. & TWENTYMAN, P.R. (1991). Retention of activity by selected anthracyclines in a multidrug resistant human large cell lung carcinoma line without P-glycoprotein hyperexpression. *Br. J. Cancer*, **63**, 351–357.

DEAN, N.M., KANEMITSU, M. & BOYNTON, A.L. (1989). Effects of the tyrosine-kinase inhibitor genistein on DNA synthesis and phospholipid-derived second messenger generation in mouse 10T1/2 fibroblasts and rat liver T51B cells. *Biochem. Biophys. Res. Commun.*, **165**, 795–801.

DE JONG, S., ZIJLSTRA, J.G., DE VRIES, E.G.E. & MULDER, N.H. (1990). Reduced DNA topoisomerase II activity and drug-induced cleavage activity in an adriamycin-resistant human small cell lung carcinoma cell line. *Cancer Res.*, **50**, 304–309.

DE LANGE, J.H.M., SCHIPPER, N.W., SCHUURHUIS, G.J., VAN HEIJNINGEN, Th.H.M., PINEDO, H.M., LANKELMA, J. & BAAK, J.P.A. (1992). Quantification by laser scan microscopy of intracellular doxorubicin distribution. *Cytometry*, **13**, 571–576.

ENDICOTT, J.A. & LING, V. (1989). The biochemistry of P-glycoprotein-mediated multidrug resistance. *Annu. Rev. Biochem.*, **58**, 137–171.

FINE, R.L., PATEL, J. & CHABNER, B.A. (1988). Phorbol esters induce multidrug resistance in human breast cancer cells. *Proc. Natl Acad. Sci, USA*, **85**, 582–586.

GERVASONI, J.E. Jr, FIELDS, S.Z., KRISHNA, S., BAKER, M.A., ROSADO, M., THURAISAMY, K., HINDENBURG, A.A. & TAUB, R.N. (1991). Subcellular distribution of daunorubicin in P-glycoprotein-positive and -negative drug-resistant cells lines using laser-assisted confocal microscopy. *Cancer Res.*, **51**, 4955–4963.

HONMA, Y., OKABE-KADO, J., KASUKABE, T., HOZUMI, M., KODAMA, H., KAJIGAYA, S., SUDA, T. & MIURA, Y. (1992). Herbimycin A, an inhibitor of tyrosine kinase, prolongs survival of mice inoculated with myeloid leukemia C1 cells with high expression of v-*abl* tyrosine kinase. *Cancer Res.*, **52**, 4017–4020.

KUIPER, C.M., BROXTERMAN, H.J., BAAS, F., SCHUURHUIS, G.J., HAISMA, H.J., SCHEFFER, G.L., LANKELMA, J. & PINEDO, H.M. (1990). Drug transport variants without P-glycoprotein overexpression from a squamous-lung-cancer cell line after selection with doxorubicin. *J. Cell. Pharmacol.*, **1**, 35–41.

LAI, S.-L., GOLDSTEIN, L.J., GOTTESMAN, M.M., PASTAN, I., TSAI, C.-M., JOHNSON, B.E., MULSHINE, J.L., IHDE, D.C., KAYSER, K. & GAZDAR, A.F. (1989). MDR1 gene expression in lung cancer. *J. Natl Cancer Inst.*, **81**, 1144–1150.

LINN, S.C., GIACCONE, G., VAN KALKEN, C.K. & PINEDO, H.M. (1992). P-glycoprotein mediated multidrug resistance and its clinical relevance in cancer treatment. *Forum*, **2**, 642–657.

MA, L., MARQUARDT, D., TAKEMOTO, L. & CENTER, M.S. (1991). Analysis of P-glycoprotein phosphorylation in HL60 cells isolated for resistance to vincristine. *J. Biol. Chem.*, **266**, 5593–5599.

MARKOVITS, J., LINASSIER, C., FOSSÉ, P., COUPRIE, J., PIERRE, J., JACQUEMIN-SABLON, A., SAUCIER, J.-M., LE PECQ, J.-B. & LARSEN, A.K. (1989). Inhibitory effects of the tyrosine kinase inhibitor genistein on mammalian DNA topoisomerase II. *Cancer Res.*, **49**, 5111–5117.

MARQUARDT, D., MCCRONE, S. & CENTER, M.S. (1990). Mechanisms of multidrug resistance in HL60 cells: detection of resistance-associated proteins with antibodies against synthetic peptides that correspond to the deduced sequence of P-glycoprotein. *Cancer Res.*, **50**, 1426–1430.

MCGRATH, T., LATOUD, C., ARNOLD, S.T., SAFA, A.R., FELSTED, L. & CENTER, M.S. (1989). Mechanisms of multidrug resistance in HL60 cells: analysis of resistance associated membrane proteins and levels of *mdr* gene expression. *Biochem. Pharmacol.*, **38**, 3611–3619.

NOONAN, K.E., BECK, C., HOLZMAYER, T.A., CHIN, J.E., WUNDER, J.S., ANDRULIS, I.L., GAZDAR, A.F., WILLMAN, C.L., GRIFFITH, B., VON HOFF, D.D. & RONINSON, I.B. (1990). Quantitative analysis of *mdr*1 (multidrug resistance) gene expression in human tumors by polymerase chain reaction. *Proc. Natl Acad. Sci, USA*, **87**, 7160–7164.

NOOTER, K., SONNEVELD, P., OOSTRUM, R., HERWEIJER, H., HAGENBEEK, T. & VALERIO, D. (1990). Overexpression of the *mdr*1 gene in blast cells from patients with acute myelocytic leukemia is associated with decreased anthracycline accumulation that can be restored by cyclosporin-A. *Int. J. Cancer*, **45**, 263–268.

SCHEPER, R.J., BROXTERMAN, H.J., SCHEFFER, G.L., KAAIJK, P., DALTON, W.S., VAN HEIJNINGEN, T.H.M., VAN KALKEN, C.K., SLOVAK, M.L., DE VRIES, E.G.E., VAN DER VALK, P., MEIJER, C.J.L.M. & PINEDO, H.M. (1993). Overexpression of a Mr 110,000 vesicular protein in non-P-glycoprotein-mediated multidrug resistance. *Cancer Res.*, **53**, 1475–1479.

SCHUURHUIS, G.J., BROXTERMAN, H.J., CERVANTES, A., VAN HEIJNINGEN, T.H.M., DE LANGE, J.H.M., BAAK, J.P.A., PINEDO, H.M. & LANKELMA, J. (1989). Quantitative determination of factors contributing to doxorubicin resistance in multidrug-resistant cells. *J. Natl Cancer Inst.*, **81**, 1887–1892.

SCHUURHUIS, G.J., PINEDO, H.M., BROXTERMAN, H.J., VAN KALKEN, C.K., KUIPER, C.M. & LANKELMA, J. (1990). Differential sensitivity of multi-drug-resistant and -sensitive cells to resistance-modifying agents and the relation with reversal of anthracycline resistance. *Int. J. Cancer*, **46**, 330–336.

SCHUURHUIS, G.J., BROXTERMAN, H.J., DE LANGE, J.H.M., PINEDO, H.M., VAN HEIJNINGEN, T.H.M., KUIPER, C.M., SCHEFFER, G.L., SCHEPER, R.J., VAN KALKEN, C.K., BAAK, J.P.A. & LANKELMA, J. (1991). Early multidrug resistance, defined by changes in intracellular doxorubicin distribution, independent of P-glycoprotein. *Br. J. Cancer*, **64**, 857–861.

SKOVSGAARD, T. & NISSEN, N.I. (1982). Membrane transport of anthracyclines. *Pharmacol. Ther.*, **18**, 293–311.

SLOVAK, M.L., HOELTGE, G.A., DALTON, W.S. & TRENT, J.M. (1988). Pharmacological and biological evidence for differing mechanisms of doxorubicin resistance in two human tumor cell lines. *Cancer Res.*, **48**, 2793–2797.

SLOVAK, M.L., HO, J., DEELEY, R.G. & COLE, S.P.C. (1993). Localization of a novel multidrug resistance associated gene on two non-P-glycoprotein mediated doxorubicin-selected cells. *Proc. Am. Ass. Cancer Res.*, **34**, 23.

TAKEDA, Y., NISHIO, K., SUGIMOTO, Y., KASAHARA, K., KUBO, S., FUJIWARA, Y., NIITANI, H. & SAIJO, N. (1991). Establishment of a human leukemia subline resistant to the growth-inhibitory effect of 12-*O*-tetradecanoylphorbol 13-acetate (TPA) and showing non-P-glycoprotein-mediated multi-drug resistance. *Int. J. Cancer*, **48**, 931-937.

TAKEDA, Y., NISHIO, K., MORIKAGE, T., KUBOTA, N., KOJIMA, A., KUBO, S., FUJIWARA, Y., NIITANI, H. & SAIJO, N. (1992). Reversal of multidrug resistance by genistein in non-P-glycoprotein mediated multidrug-resistant cell line (K562/TPA). *Proc. Amer. Ass. Cancer Res.*, **33**, 476.

TAYLOR, C.W., DALTON, W.S., PARRISH, P.R., GLEASON, M.C., BELLAMY, W.T., THOMPSON, F.H., ROE, D.J. & TRENT, J.M. (1991). Different mechanisms of decreased drug accumulation in doxorubicin and mitoxantrone resistant variants of the MCF7 human breast cancer cell line. *Br. J. Cancer*, **63**, 923-929.

VERSANTVOORT, C.H.M., BROXTERMAN, H.J., FELLER, N., DEKKER, H., KUIPER, C.M. & LANKELMA, J.C. 1992*a*). Probing daunorubicin accumulation defects in non-P-glycoprotein expressing multidrug-resistant cell lines using digitonin. *Int. J. Cancer*, **50**, 906-911.

VERSANTVOORT, C.H.M., BROXTERMAN, H.J., PINEDO, H.M., FELLER, N., KUIPER, C.M. & LANKELMA, J. (1992*b*). Energy-dependent processes involved in reduced drug accumulation in multidrug-resistant human lung cancer cell lines without P-glycoprotein expression. *Cancer Res.*, **52**, 17-23.

VERSANTVOORT, C.H.M., TWENTYMAN, P.R., BARRAND, M.A., LANKELMA, J., PINEDO, H.M. & BROXTERMAN, H.J. (1992*c*). Overexpression of 110 and 190 kD proteins in cancer cells may be involved in drug resistant phenotype. *Proc. Am. Assoc. Cancer Res.*, **33**, 456.

YONEDA, T., LYALL, R.M., ALSINA, M.M., PERSONS, P.E., SPADA, A.P., LEVITZKI, A., ZILBERSTEIN, A. & MUNDY, G.R. (1991). The antiproliferative effects of tyrosine kinase inhibitors tyrphostins on a human squamous cell carcinoma *in vitro* and in nude mice. *Cancer Res.*, **51**, 4430-4435.

YU, G., AHMAD, S., AQUINO, A., FAIRCHILD, C.R., TREPEL, J.B., OHNO, S., SUZUKI, K., TSURUO, T., COWAN, K.H. & GLAZER, R.I. (1991). Transfection with protein kinase Cα confers increased multidrug resistance to MCF-7 cells expressing P-glycoprotein. *Cancer Commun.*, **3**, 181-189.

YUSA, K. & TSURUO, T. (1989). Reversal mechanism of multidrug resistance by verapamil: direct binding of verapamil to P-glycoprotein on specific sites and transport of verapamil outward across the plasma membrane of K562/ADM cells. *Cancer Res.*, **49**, 5002-5006.

QIAN, X.-D. & BECK, W.T. (1990). Binding of optically pure photaffinity analogue of verapamil, LU-49888, to P-glycoprotein from multidrug-resistant leukemic cell lines. *Cancer Res.*, **50**, 1132-1137.

ZAMAN, G.J.R., VERSANTVOORT, C.H.M., SMIT, J.J.M., EIJDEMS, E.W.H.M., DE HAAS, M., SMITH, A.J., BROXTERMAN, H.J., MULDER, N.H., DE VRIES, E.G.E, BAAS, F. & BORST, P. (1993). Analysis of the expression of *MRP*, the gene for a new putative transmembrane drug transporter, in human multidrug resistant lung cancer cell lines. *Cancer Res.*, **53**, 1747-1750.

ZIJLSTRA, J.G., DE VRIES, E.G.E. & MULDER, N.H. (1987). Multifactorial drug resistance in an Adriamycin-resistant human small cell lung carcinoma cell line. *Cancer Res.*, **47**, 1780-1784.